# Full-Length Genome Sequence of Porcine Epidemic Diarrhea Virus Strain CH/GX/2015/750A

**DOI:** 10.1128/genomeA.00361-17

**Published:** 2017-07-06

**Authors:** Yibin Qin, Bingxia Lu, Ying He, Bin Li, Qunpeng Duan, Jiaxing Liang, Zhongwei Chen, Qianlian Su, Bingfen Bi, Wu Zhao

**Affiliations:** Guangxi Key Laboratory of Veterinary Biotechnology/Department of Virology, Guangxi Veterinary Research Institute, Nanning, Guangxi, China

## Abstract

We report here the complete genome sequence of porcine epidemic diarrhea virus (PEDV) strain CH/GX/2015/750A (750A), which was isolated from a suckling piglet with watery diarrhea in Guangxi, China. The isolate is genetically close to other recent Chinese variant PEDVs and distinct from the classical PEDVs.

## GENOME ANNOUNCEMENT

Porcine epidemic diarrhea virus (PEDV) is a member of the *Alphacoronavirus* genus in the family *Coronaviridae*; it is an enveloped, single-stranded, positive-sense RNA virus that causes watery diarrhea, vomiting, dehydration, and high mortality in suckling piglets (1, 2). PED was initially reported in British swine herds in the early 1970s (3, 4), and the prototype PEDV strain CV777 was described in 1978 (5). Since then, outbreaks of PEDV infections have been reported in multiple swine-producing countries, especially in Europe and Asia (6–10). In North America, PEDV was identified for the first time in the United States in May 2013 (11).

Since late 2010, outbreaks of PED, which were caused by a variant PEDV, have been observed in swine farms in China, even though some swine populations were immunized with vaccines derived from PEDV strain CV777 (12–15). PEDV strain CH/GX/2015/750A (750A) was isolated from the intestinal contents of a suckling piglet with acute diarrhea on a commercial swine farm in Guangxi Province in 2015. Typical cytopathic effect (CPE), characterized by cell fusion, syncytium formation, and eventual detachment, could be observed in 750A-infected Vero cells after three blind passages. To understand the genetic characterization and evolutionary characteristics of 750A, the complete genome was sequenced using next-generation sequencing technology, and the data were assembled using Velvet (version 1.2.10) software based on known PEDV sequences.

The complete genomic RNA of the isolate is 28,038 nucleotides (nt) in length, excluding the 3′-poly(A) tail. The genomic organization of the isolate is similar to what was previously described (12, 13, 15) and includes a 5′ untranslated region (5′ UTR) (nt 1 to 292), open reading frame 1a (ORF1a) (nt 293 to 12616) and ORF1b (nt 12616 to 20637) encoding a replicase polyprotein, spike (S) gene (nt 20634 to 24794), ORF3 (nt 24794 to 25468), envelope (E) gene (nt 25449 to 25679), membrane (M) gene (nt 25687 to 26367), nucleocapsid (N) gene (nt 26379 to 27704), and 3′ UTR (nt 27705 to 28038). Compared to the classical strain CH/S, virulent strain DR13, and the prototype PEDV strain CV777 (16–18), the S protein of strain 750A has two insertion regions (^59^QGVN^62^ and ^140^N) and one deletion region (^161^GK^162^) in the N-terminal region, and these insertions and the deletion were also observed in other Chinese strains (12, 13, 15) and the U.S. virulent strains (19, 20).

The complete genome of PEDV strain 750A has a high nucleotide identity of 98.2% to 99.8% with other PEDV strains circulating in Asia, North America, and Europe in recent years, with the highest nucleotide identity (99.8%) with Chinese strain YC2014 (21). However, 750A has a relatively low nucleotide identity (96.8% to 97.7%) with classical strains and vaccine strains (CV777 and DR13) in Asia, which might explain the incomplete protection efficiency of the vaccination.

The genome sequence reported in this study will promote a better understanding of the molecular pathogenesis and genetic diversity of PEDV.

### Accession number(s).

The complete genome sequence of PEDV strain CH/GX/2015/750A has been deposited in GenBank under the accession no. KY793536.
